# Diagnostic utility of basal luteinizing hormone in evaluating central precocious puberty

**DOI:** 10.1210/jendso/bvag131

**Published:** 2026-06-16

**Authors:** Vittorio Ferrari, Daniela Elleri, Jennifer Roach, Mike Crane, Rod Thomas Mitchell

**Affiliations:** Pediatric Unit, IRCCS Azienda Ospedaliero-Universitaria di Bologna, Bologna 40138, Italy; Department of Paediatric Endocrinology, Royal Hospital for Children and Young People, Edinburgh EH16 4TJ, UK; Department of Paediatric Endocrinology, Royal Hospital for Children and Young People, Edinburgh EH16 4TJ, UK; Department of Clinical Biochemistry, Royal Infirmary of Edinburgh, Edinburgh EH16 4SA, UK; Department of Paediatric Endocrinology, Royal Hospital for Children and Young People, Edinburgh EH16 4TJ, UK; Centre for Reproductive Health, Institute of Regeneration and Repair, University of Edinburgh, Edinburgh EH16 4UU, UK

**Keywords:** basal luteinizing hormone, GnRH stimulation test, central precocious puberty, early puberty, HPG axis activation, pubertal assessment

## Abstract

**Background:**

Basal luteinizing hormone (bLH) is increasingly proposed as a surrogate marker of hypothalamic–pituitary–gonadal activation, potentially reducing the need for gonadotropin-releasing hormone (GnRH) testing in children with suspected central precocious puberty (CPP). However, diagnostic thresholds remain assay-dependent, and values below the detection limit complicate interpretation.

**Objective:**

This study aims to assess the diagnostic performance of bLH in predicting GnRH test response in patients evaluated for suspected CPP, using a censoring-aware approach for values below the assay limit of detection (LOD).

**Methods:**

We retrospectively analyzed 258 first-time GnRH stimulation tests performed for suspected CPP between 2011 and 2025 at the Royal Hospital for Children and Young People, Edinburgh. Basal luteinizing hormone was measured in fasting early-morning samples collected between 08:00 and 10:00 Am immediately before GnRH administration, with Abbott Architect (LOD 0.5 U/L) or Roche Cobas (LOD 0.3 U/L). Values below the assay-specific LOD were treated as left-censored observations and handled with parametric regression on order statistics. Receiver operating characteristic analyses were performed separately by sex.

**Results:**

Basal luteinizing hormone showed good accuracy for predicting a pubertal GnRH response, with an AUC of 0.80. The optimal threshold was 0.5 U/L (sensitivity 0.61, specificity 0.97), while a bLH ≥ 1.0 U/L achieved 100% specificity and positive predictive value. Sex-stratified analyses showed similar performance in girls and boys, although discrimination was higher in boys. Combined models including bLH and sex steroids did not materially improve discrimination in girls, whereas the model combining bLH and testosterone showed strong performance in boys (AUC 0.88).

**Conclusion:**

Early-morning bLH provides clinically useful information for the assessment of suspected CPP. Higher values can reliably predict a positive GnRH response, supporting its use as a first-line test and a more selective use of GnRH stimulation testing, whereas low or indeterminate values still require careful clinical interpretation.

Pubertal onset is a developmental process involving physical, hormonal, and psychological changes that occur over several years [1]. In girls, it is defined by the appearance of breast buds between 8 and 13 years and in boys by testicular enlargement ≥ 4 mL after 9 years [1, 2]. It is driven by reactivation of the hypothalamic–pituitary–gonadal (HPG) axis, leading to increased secretion of gonadotropins and sex steroids, with subsequent development of secondary sexual characteristics, growth acceleration, and bone maturation [1, 3-5].

Precocious puberty (PP) is defined as the appearance of secondary sexual characteristics before age 8 years in girls and age 9 years in boys. Its most common form, central precocious puberty (CPP), results from premature activation of the HPG axis and is more frequent in females [6, 7]. Isolated early breast development may instead reflect idiopathic premature thelarche, a usually benign condition that may occasionally progress to CPP and therefore requires follow-up [8, 9].

The diagnostic evaluation of pubertal disorders is based on clinical examination, growth velocity, Tanner staging, and bone age assessment. Among auxological markers, advancement of bone age by more than 2 standard deviations has been proposed as predictive of CPP [10]. Imaging may provide additional information, particularly in girls, where pelvic ultrasonography is used to assess uterine and ovarian size, although considerable overlap between prepubertal and early pubertal findings limits its accuracy [5, 11].

The gonadotropin-releasing hormone (GnRH) stimulation test remains widely used as the reference test for confirming central activation of the HPG axis in suspected CPP. A stimulated luteinizing hormone (LH) peak > 5 U/L supports activation of the HPG axis, while an LH/FSH peak ratio > 1 may help identify more rapidly progressive forms [5, 12-14]. However, the test is invasive, time-consuming, and costly and requires repeated blood sampling [5, 14-16]. Its sensitivity may be lower in the earliest phases of PP, and interpretation criteria vary across studies, with no international consensus on optimal cutoff values [16-18].

Given these limitations, several studies have explored alternative markers of pubertal onset, particularly basal LH (bLH) as a practical indicator of HPG axis activation [5, 18-21]. Although bLH is now considered a useful predictor of GnRH stimulation test response, reported results vary across studies according to assay characteristics and study design, and universal cutoffs remain undefined [19-25]. In addition, values below the limit of detection have usually been handled using pragmatic approaches that may affect the accuracy of diagnostic estimates.

In this context, the aim of this study was to examine the ability of early-morning bLH to predict GnRH stimulation test response in patients with suspected CPP, using a more refined approach for values below the limit of detection, and to explore the potential implications of its use for resource utilization through a reduction in stimulation testing when appropriate.

## Materials and methods

### Study design

This retrospective single-center study was conducted at the Pediatric Endocrinology Department of the RHCYP, Edinburgh, United Kingdom. Consecutive patients of both sexes referred for suspected CPP between January 2011 and August 2025 were evaluated and data were obtained from TrakCare™ patient information system. For each patient, only the first GnRH stimulation test performed during the study period was considered, in order to avoid potential bias from prior testing that could have influenced subsequent hormonal dynamics. Tests were excluded if they failed because of cannulation errors, sample coagulation, or missing laboratory results. Eligibility criteria for suspected CPP were breast development (Tanner stage B2) before 8 years of age in girls and testicular volume ≥ 4 mL (Tanner stage G2) before 9 years of age in boys. Patients were excluded if they had peripheral forms of PP, primary gonadal dysfunction, chronic illness, a history of oncological treatment, or current/recent exposure to medications known to interfere with HPG axis function.

### Clinical, auxological, and imaging evaluation

At the time of the GnRH stimulation test, height and weight were measured by trained staff using standardized procedures, and BMI was calculated as weight (kg)/height^2^ (m). Anthropometric values were expressed as standard deviation scores (SDS) according to the UK-WHO growth reference charts (WHO 0-4 years [26]; UK90 4-20 years [27]). Pubertal development (breast, axillary, and pubic hair) was staged according to Tanner and Whitehouse criteria [28], and testicular volume was assessed using a Prader orchidometer.

Bone age was assessed from left-hand and wrist radiographs according to the Tanner-Whitehouse 3 (TW3) method [29], and the difference between bone age and chronological age (BA–CA) was calculated to estimate advancement in skeletal maturation. In girls with a distended bladder, transabdominal pelvic ultrasound was performed by pediatric radiologists using high-frequency probes (3.5-6.5 MHz). Uterine diameters (longitudinal, transverse, and anteroposterior) were measured, and uterine volume was calculated using the ellipsoid formula (length × width × depth × 0.5233).

### Hormonal evaluation

Endocrine evaluation included basal serum measurements of LH, FSH, 17β-estradiol, and testosterone, obtained after overnight fasting in the early morning, between 08:00 and 10:00 Am, immediately before GnRH administration. Prior to October 2021, LH, FSH, and estradiol were measured using the Abbott Architect platform (chemiluminescent microparticle immunoassay, CMIA): Architect LH Reagent Kit (Abbott, Cat# 2P40-25, RRID:AB_2813909), Architect FSH Reagent Kit (Abbott, Cat# 7K75-25, RRID:AB_2813910), and Architect Estradiol Reagent Kit (Abbott, Cat# 7K72-25, RRID:AB_2813911). The lower limits of reporting were <0.5 U/L for LH and FSH and <50 pmol/L for estradiol. From October 2021 onwards, these analytes were measured using Roche Elecsys electrochemiluminescence immunoassays (ECLIA) on the Cobas platform: Elecsys LH (Roche, Cat# 07027575190, RRID:AB_2920601), Elecsys FSH (Roche, Cat# 07027346190, RRID:AB_2920600), and Elecsys Estradiol III (Roche, Cat# 07027249190, RRID:AB_2920599). The lower limits of reporting were <0.3 U/L for LH and FSH and <50 pmol/L for estradiol. Testosterone concentrations were consistently measured throughout the study period using an in-house LC/MS assay, with a lower limit of reporting of <0.3 nmol/L. All patients underwent a GnRH stimulation test. Intravenous synthetic gonadorelin acetate (50-100 μg, maximum dose 100 μg) was administered, with blood samples collected at baseline, 20, and 60 minutes for LH and FSH determination. A pubertal response was defined as a peak LH concentration ≥5 U/L; an LH/FSH ratio > 1 was considered supportive evidence of HPG axis activation. The highest LH and FSH measurements of the 2 samples taken after GnRH injection were denoted as the peak values.

### Statistical analysis

Continuous variables were summarized as mean ± standard deviation (SD). Anthropometric variables were also expressed as SDS. For comparisons between groups, we used Student's t test when the data were normally distributed and the Mann–Whitney U test when data did not follow a normal distribution. A 2-sided *P* value below .05 was considered to indicate statistical significance.

The reference standard was the LH peak after GnRH stimulation (maximum post-stimulus value), with a positive test defined as LH peak ≥ 5 U/L. The aim of the analysis was to assess how well bLH could predict this outcome.

Because many bLH results were reported below the assay's limit of detection (LOD = 0.5 or 0.3 U/L, depending on the study period), these values were treated as left-censored, meaning that the true concentration was known to be below the assay-specific LOD but could not be quantified exactly. We used parametric regression on order statistics (ROS) under a log-normal assumption, and we checked this assumption with the Shapiro–Wilk test and Q–Q plots of log-transformed values. Each result was assigned the correct LOD according to the assay used (Abbott Architect before October 2021; Roche Cobas after October 2021). To test robustness, we repeated the analyses replacing values below LOD with either half the detection limit (LOD/2) or the detection limit itself (=LOD).

For each approach (ROS, LOD/2, =LOD), we built receiver operating characteristic (ROC) curves to evaluate how well bLH predicted the GnRH test result. We calculated the area under the curve (AUC) overall and by sex, and we reported sensitivity, specificity, positive predictive value (PPV), and negative predictive value (NPV) at clinically relevant thresholds (0.5, 1.0, 1.5 U/L, and the Youden cutoff). To estimate uncertainty for these measures, we used nonparametric bootstrap resampling with 1000 replicates and reported 95% confidence intervals (CIs). In addition, to derive rule-out and rule-in thresholds for bLH, we fitted a logistic regression model predicting the probability of a pubertal response as a function of bLH. The bLH values corresponding to predicted probabilities of 5% and 95% were taken as rule-out and rule-in cutoffs, respectively, defining a diagnostic gray zone in between.

Because the assay platform changed during the study, we repeated the analyses restricted to the Roche cohort (LOD 0.3 U/L). As reported in the “Results” section, the findings were consistent with the main analysis, indicating that the platform change did not materially affect diagnostic performance.

Finally, we evaluated ancillary markers (estradiol in girls, testosterone in boys, BA–CA, and uterine volume in girls) to assess their individual discriminative ability. For each variable, we constructed ROC curves and calculated the AUC, the Youden cutoff, sensitivity, and specificity.

All ROC analyses for bLH used the full sample after <LOD handling (ROS/LOD/2/=LOD); ancillary markers were analyzed using a complete-case approach, restricted to subjects with available data.

To assess whether combining gonadotropins and steroidal markers could improve diagnostic discrimination, we built multivariable logistic regression models including bLH together with basal estradiol in girls and basal testosterone in boys. The predicted probabilities from these models were used to construct combined ROC curves, from which the AUC and the Youden index were derived. For each model, we also reported the optimal probability threshold, sensitivity, and specificity.

All analyses were conducted in R (version 4.5.1, R Foundation for Statistical Computing, Vienna, Austria).

## Results

A total of 258 first-time GnRH stimulation tests performed for suspected CPP between January 2011 and August 2025 at the Royal Hospital for Children & Young People, Edinburgh, were included. Demographic and auxological characteristics at the time of GnRH stimulation testing are summarized in Table 1.

**Table 1:** Anthropometric characteristics of patients at the time of GnRH stimulation testing

Sex	N (%)	Age (years)	Height (cm, SDS)	Weight (kg, SDS)	BMI (kg/m^2^, SDS)
♀	226/258 (87.6%)	7.43 ± 1.36	130.22 ± 12.16 (1.22 ± 1.50)	32.637 ± 9.599 (1.38 ± 1.25)	19.32 ± 6.59 (1.16 ± 1.30)
♂	32/258 (12.4%)	8.43 ± 0.81	137.23 ± 11.04 (1.28 ± 1.65)	38.536 ± 10.337 (1.73 ± 1.31)	20.33 ± 3.46 (1.75 ± 1.13)

Abbreviations: ♀, females; ♂, males.

Basal LH values included a high proportion of left-censored measurements, with 185/258 (71.7%) falling below the assay limit of detection; the assay LOD changed during the study period from 0.5 to 0.3 U/L (n = 166 and n = 19, respectively). The assumption of log-normality was supported for log-transformed bLH values (Shapiro–Wilk *P* = .204), with no major deviation observed on Q–Q plots (Fig. S1 [30]). Accordingly, <LOD values were handled primarily using parametric ROS, assuming a log-normal distribution, and all estimates were repeated using =LOD and LOD/2 substitution as sensitivity analyses.

**Figure 1:**
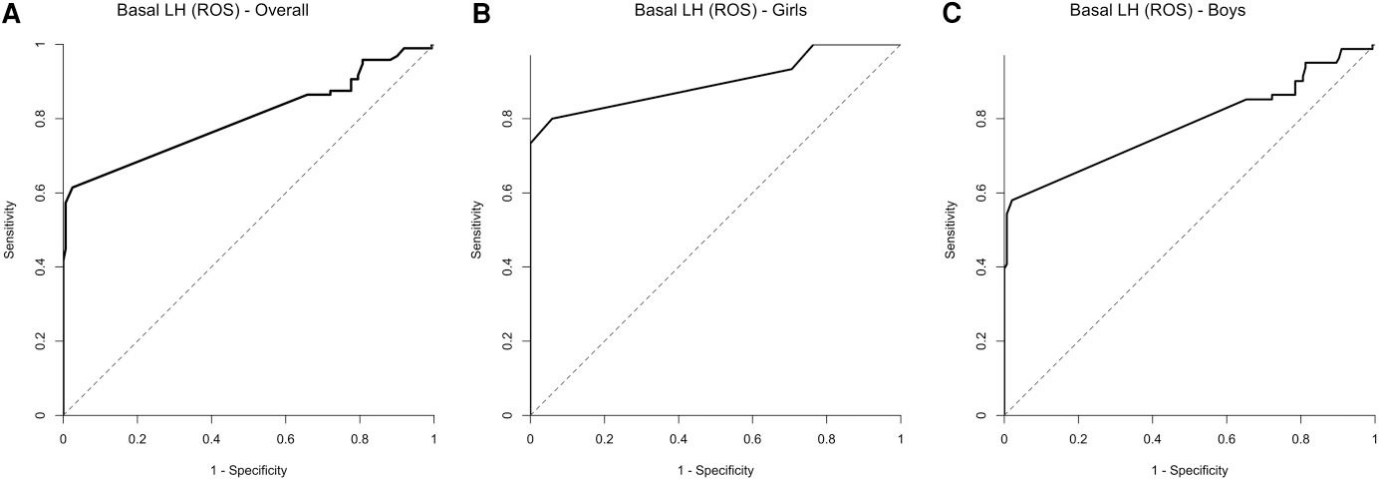
ROC curves for bLH. ROC curves for bLH as a predictor of GnRH test response in patients evaluated for suspected CPP. Values below the assay limit of detection were handled using regression on order statistics. (A) Overall cohort, (B) girls, and (C) boys. Abbreviations: CPP, central precocious puberty; GnRH, gonadotropin-releasing hormone; LH, luteinizing hormone; ROC, receiver operating characteristic.

Using ROS, bLH showed good overall discriminatory ability for pubertal response to the GnRH test (Fig. 1A). Results obtained with alternative < LOD handling approaches were broadly comparable, supporting the robustness of the main findings (Fig. S2A and S2B [30]). Sex-stratified analyses showed a similar pattern in girls and boys, although discrimination was higher in boys (Fig. 1B and 1C; Fig. S3A-S3D [30]). The full estimates for the overall and sex-stratified analyses based on ROS are summarized in Table 2, while corresponding results obtained using alternative < LOD handling methods are reported in Table S1 [30].

**Table 2:** Diagnostic performance of basal LH

Analysis	Subgroup	AUC	Optimal threshold	Sensitivity	Specificity
ROS	Total	0.80	0.50	61%	97%
	♀	0.78	0.50	58%	98%
	♂	0.89	0.50	80%	94%

AUC, Youden-optimal threshold, sensitivity, and specificity for basal LH estimated using regression on order statistics, overall, and by sex. Alternative approaches to <LOD handling are shown in Table S1 [30].

Abbreviations: AUC, area under the curve; LH, luteinizing hormone; ♀, females; ♂, males.

Fixed cutoff analyses based on ROS showed that 0.5 U/L provided the best balance between sensitivity and specificity, whereas 1.0 U/L was the lowest threshold achieving 100% specificity; increasing the threshold further to 1.5 U/L did not improve specificity but further reduced sensitivity (Table 3). Exploratory rule-out and rule-in zones were also delineated in relation to GnRH test outcome. A rule-out threshold ≤ 0.24 U/L identified virtually no pubertal responders, whereas values ≥ 1.01 U/L distinguished only GnRH responders. Intermediate values defined a diagnostic gray zone in which bLH alone did not allow reliable classification.

**Table 3:** Diagnostic performance of basal LH at fixed cutoff values

Cutoff (U/L)	Sensitivity	Specificity	PPV	NPV
0.5	74%	89%	92%	82%
1.0	41%	100%	100%	74%
1.5	24%	100%	100%	69%

Sensitivity, specificity, positive predictive value, and negative predictive value at basal LH cutoffs of 0.5, 1.0, and 1.5 U/L.

Abbreviations: LH, luteinizing hormone; NPV, negative predictive value; PPV, positive predictive value.

**Figure 2:**
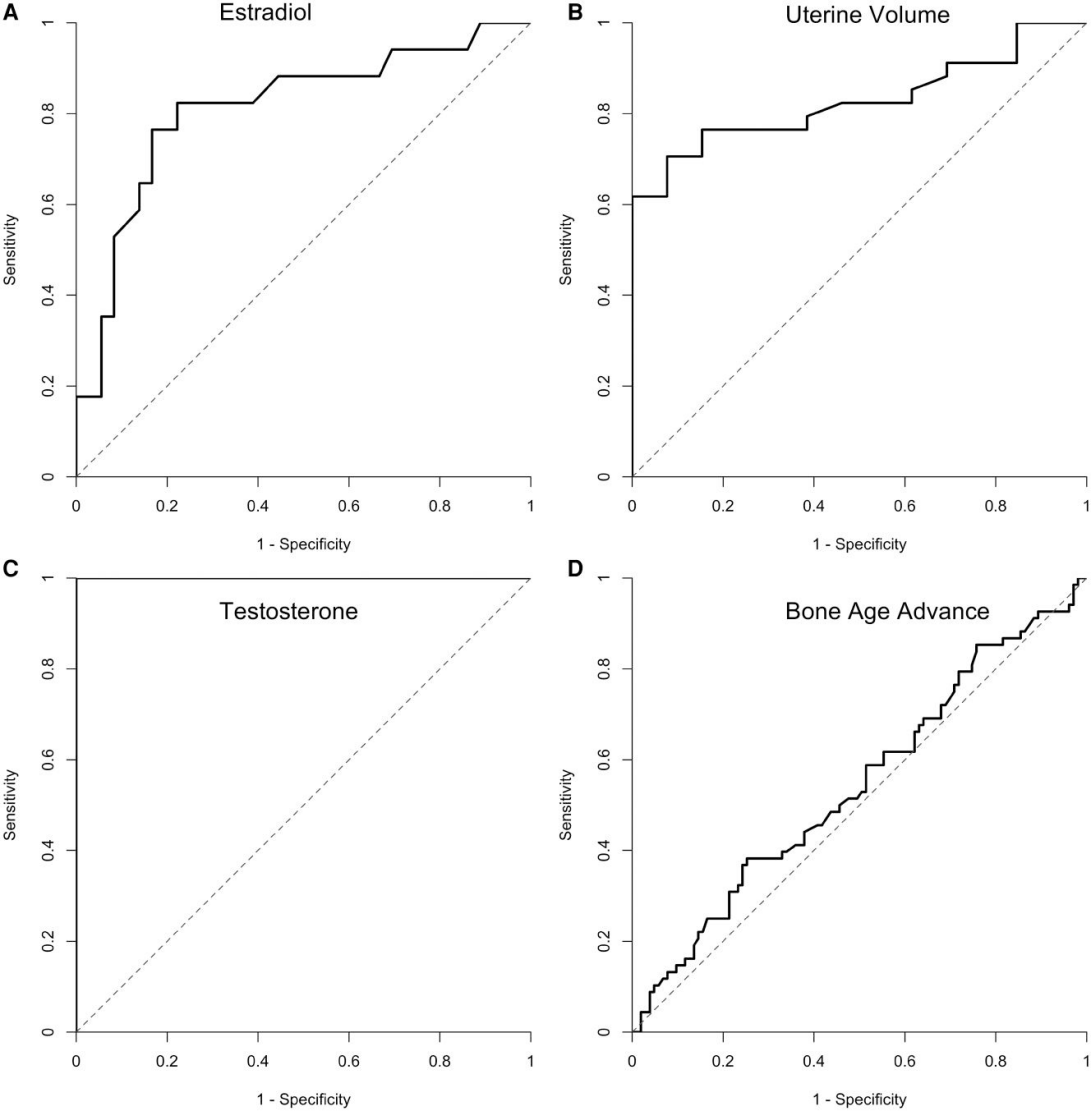
ROC curves for ancillary markers. ROC curves for ancillary markers as predictors of GnRH test response in patients evaluated for suspected CPP. Panel (A) shows basal estradiol in girls, panel (B) uterine volume in girls, panel (C) testosterone in boys, and panel (D) bone age advancement in the overall cohort. Abbreviations: CPP, central precocious puberty; GnRH, gonadotropin-releasing hormone; ROC, receiver operating characteristic.

Among ancillary markers, estradiol in girls showed modest discriminatory performance, whereas uterine volume showed good predictive accuracy. In boys, basal testosterone showed excellent discriminatory performance, while bone age advancement performed poorly both overall and after sex stratification. Full estimates for ancillary markers are summarized in Table 4, with the corresponding ROC curves shown in Fig. 2A-2D and Fig. S4A and S4B [30].

**Table 4:** Diagnostic performance of ancillary markers

Marker	Subgroup	N	AUC	Optimal threshold	Sensitivity	Specificity
Estradiol	♀	224	0.66	52.5 pmol/L	39%	93%
Uterine volume	♀	53	0.81	2.5 mL	82%	78%
Testosterone	♂	29	1.00	0.55 nmol/L	100%	100%
Bone age advancement	Overall	171	0.54	1.91 years	38%	74%
	♀	152	0.53	1.91 years	36%	75%
	♂	19	0.62	0.72 years	75%	54%

For each marker, the table reports subgroup, sample size, AUC, Youden-optimal threshold, sensitivity, and specificity. Estradiol and uterine volume were evaluated in girls, testosterone in boys, and bone age advancement overall and after sex stratification.

Abbreviations: AUC, area under the curve; ♀, female; ♂, male.

**Figure 3:**
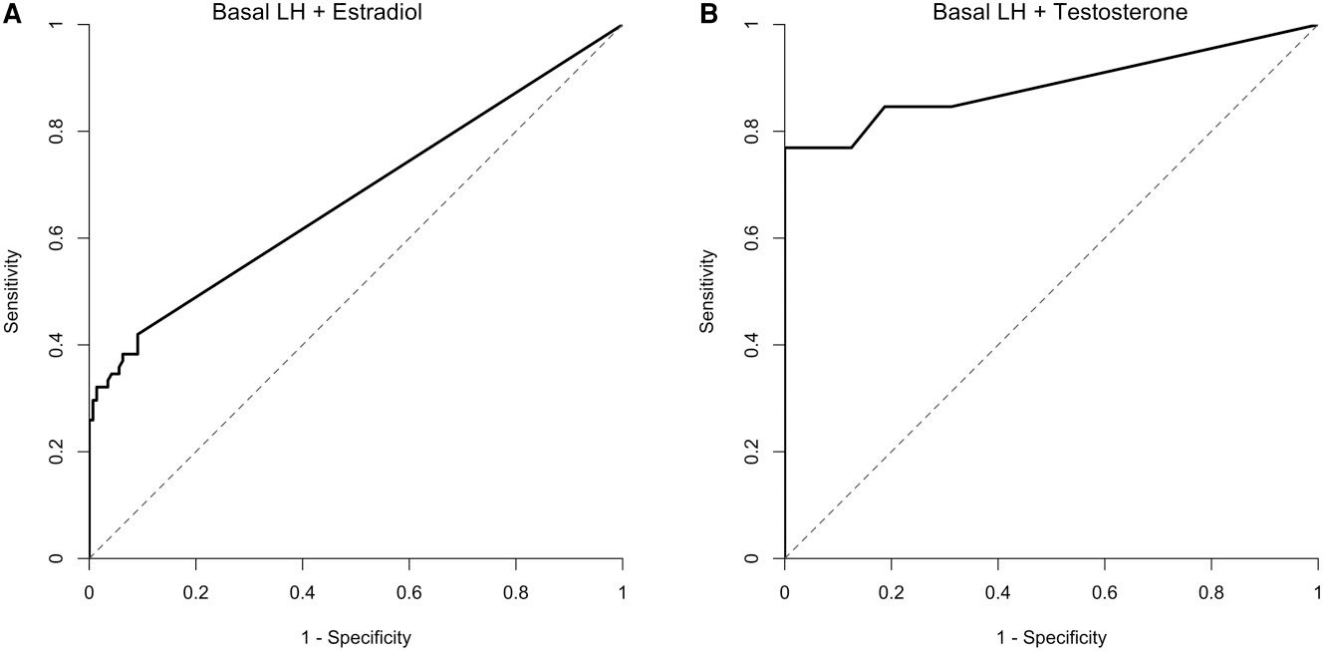
ROC curves for combined models. ROC curves for multivariable logistic regression models combining bLH with sex steroids in patients evaluated for suspected CPP. Panel (A) shows the model including bLH and estradiol; panel (B) the model including bLH and testosterone. Abbreviations: CPP, central precocious puberty; GnRH, gonadotropin-releasing hormone; LH, luteinizing hormone; ROC, receiver operating characteristic.

Multivariable models were also evaluated to assess the combined performance of bLH with sex steroid markers. In girls, the model including bLH and estradiol showed moderate discriminatory ability, whereas in boys, the model combining bLH and testosterone showed strong discriminatory ability. Detailed results for both combined models are summarized in Table 5, and the corresponding ROC curves are shown in Fig. 3A and 3B.

**Table 5:** Diagnostic performance of combined models including basal LH and sex steroid markers

Model	N	GnRH+/N (%)	AUC (95% CI)	Optimal probability threshold	Sensitivity	Specificity	β for bLH	β for second marker
bLH + estradiol	224	81/224 (36.2%)	0.68 (0.62-0.74)	0.29	42%	91%	−.002	+.034
bLH + testosterone	29	13/29 (44.8%)	0.88 (0.75-1.00)	0.60	77%	100%	−1.391	+8.512

Multivariable logistic regression models combining basal LH with estradiol in girls or testosterone in boys. For each model, the table reports sample size, number and proportion of GnRH responders, AUC, optimal predicted-probability threshold, sensitivity, specificity, and regression coefficients for basal LH and the second marker.

Abbreviations: AUC, area under the curve; GnRH, gonadotropin-releasing hormone; LH, luteinizing hormone.

To address the mid-study change of assay platform, we repeated the analyses restricted to patients measured with the Roche Cobas system (LOD 0.3 U/L). Basal luteinizing hormone retained good discriminative ability (AUC 0.90), with an optimal threshold of 0.5 U/L yielding sensitivity 82% and specificity 92% (Fig. S5 [30]).

## Discussion

In this single-center cohort of children evaluated for suspected CPP, early-morning bLH showed good diagnostic performance for identifying HPG axis activation. Basal luteinizing hormone values ≥ 1.0 U/L had clear rule-in value, whereas lower values did not reliably exclude a pubertal GnRH response, consistent with the physiology of early pubertal activation, when LH secretion remains pulsatile and may be underestimated in later daytime basal samples [21, 23, 25].

### Interpreting basal LH thresholds in clinical practice

In our cohort, a bLH cutoff of 0.5 U/L provided the best balance between sensitivity and specificity, whereas values ≥ 1.0 U/L yielded 100% specificity and PPV for a positive GnRH response, supporting a rule-in use without the need for a stimulation test. By contrast, lower values did not reliably exclude a pubertal response, underscoring the need for cautious interpretation in early HPG axis activation. Because LH secretion shows diurnal variation during early pubertal activation, bLH should preferably be assessed in early-morning samples. Accordingly, the thresholds identified in this study should be interpreted in the context of fasting samples collected between 08:00 and 10:00 Am and may not be directly transferable to samples obtained later in the day. This pattern is consistent with previous studies, although reported thresholds vary according to assay methodology. In studies based on IMMULITE immunochemiluminometric assay (ICMA), very low cutoffs have been proposed: Pasternak et al [21] identified a threshold of 0.10 U/L with high specificity but limited sensitivity, while Li Pomi et al [31] reported a cutoff of 0.14 U/L with high sensitivity but moderate specificity. Within the same ICMA method, Harrington et al [25] proposed a dual-zone approach (≤0.2 U/L to rule out; ≥0.3 U/L to rule in), highlighting the potential for stratified decision-making in this low concentration range. Studies using time-resolved fluoroimmunoassay (TR-FIA) have generally reported somewhat higher thresholds, such as the 0.25 U/L ROC-optimal cutoff and >0.53 U/L rule-in threshold described by Cao et al [23]. These studies suggest that the diagnostic accuracy of bLH is most limited in the very early phases of pubertal activation, particularly at very low concentrations. This interpretation is further supported by the known circadian pattern of LH secretion and by evidence that early-morning sampling, commonly defined as sampling before 10:00 Am, improves the sensitivity of bLH for detecting early pubertal activation [22, 32]. Despite these differences, a consistent finding across platforms is that lower bLH values may be inconclusive in early puberty, whereas clearly elevated values provide stronger diagnostic support for central activation. In this regard, values ≥ 1.0-1.5 U/L have been repeatedly associated with near-complete specificity [33, 34], in line with our findings. Within this context, our data also showed a dual-zone pattern, with values above 1.01 U/L associated with pubertal GnRH responses and values below 0.24 U/L consistently observed in prepubertal subjects, while intermediate values remained nondiscriminatory. However, the lower threshold falls below the assay detection limit used in our current practice, limiting its applicability as a rule-out threshold in our setting.

### Combined hormonal models

The addition of basal sex steroids did not materially improve the diagnostic performance of bLH. In girls, inclusion of estradiol was associated with lower discriminatory performance than bLH alone, whereas in boys, inclusion of testosterone yielded similar accuracy to bLH alone. Overall, these findings suggest that bLH retains most of the diagnostic signal, with only a limited complementary contribution from basal sex steroids. Pasternak et al [21] found that adding FSH or the LH/FSH ratio did not improve discrimination compared with bLH alone, which remained the most reliable single basal marker for CPP. Li Pomi et al [31] proposed a sequential basal approach in which bLH ≥ 0.14 mU/mL was followed by an LH/FSH ratio ≥ 0.1, improving specificity but still relying primarily on bLH as the initial discriminator. Likewise, studies evaluating basal sex steroids separately have generally shown limited diagnostic utility in early pubertal activation, particularly for estradiol [22, 34]. Consistent with previous reports, ancillary hormonal markers appear to provide supportive diagnostic information rather than serving as a clear combined marker of HPG axis activation in CPP.

### Implications for diagnostic workflow

A practical implication of our findings is the potential reduction in GnRH stimulation testing. Adopting a rule-in threshold of bLH ≥ 1.0 U/L would have avoided approximately 15% of stimulation tests, all of them true positives. This finding is consistent with Durá-Travé et al [34], who reported PPV of 100% at the same threshold in girls, supporting the omission of stimulation testing when bLH is clearly pubertal. Overall, these findings and previous CPP studies suggest that the judicious application of high-specificity thresholds can reduce the need for stimulation testing by roughly 20-35%, or up to 75% when dual-zone algorithms are applied [19, 22, 25]. While bLH cannot fully replace the GnRH test, its appropriate use can meaningfully streamline diagnostic pathways and minimize unnecessary procedures. This potential reduction has direct practical consequences. The GnRH stimulation test requires serial blood sampling over 1 hour, constant staff supervision, and the handling of multiple samples per patient, making it a relatively demanding procedure for both healthcare providers and families. At our institution, the estimated direct cost of a single GnRH stimulation test is approximately US$840, including US$122 for GnRH, US$298 in staff costs, US$404 for Pediatric Investigation Unit use, and US$16 for blood analyses.

In our cohort, applying a high-specificity rule-in threshold would have led to a potential direct cost saving of approximately US$32 800 over the study period, with all avoided tests representing true-positive cases. This reduction translates into fewer procedures, shorter clinic occupancy, and less patient discomfort, without compromising diagnostic accuracy. Although bLH cannot fully replace stimulation testing, its use as a first-line assessment provides a realistic balance between accuracy and practicality. Table 6 summarizes the main published studies on bLH for CPP assessment.

**Table 6:** Summary of published studies evaluating the diagnostic performance of basal LH for CPP

Study (year)	Number of patients	Assay methodology	Threshold (U/L)	Sensitivity/specificity	Avoidable GnRH tests
Houk et al (2009) [19]	55	Architect CMIA; Delfia TR-FIA	0.83 (Architect), 1.05 (Delfia)	Architect: 93%/100%; Delfia: 100%/100%	Not reported
Pasternak et al (2012) [21]	80	IMMULITE ICMA	0.10	64%/95%	Not reported
Lee et al (2013) [22]	160	Roche Cobas ECLIA	0.22	69.4%/82.1%	Not reported
Harrington et al (2014) [25]	57	IMMULITE (ICMA)	≤0.1 rule-out; ≥0.3 rule-in	90.5%/100%	∼75%
Durá-Travé et al (2021) [34]	241	IMMULITE ICMA	0.1	82%/90%	Not reported
Cao et al (2021) [23]	1492	Delfia TR-FIA	0.53	44%/100%	Not reported
Li Pomi et al (2024) [31]	248	IMMULITE ICMA	0.14	90.6%/78.2%	∼22%
Baronio et al (2024) [33]	213	Roche Elecsys ECLIA	1.5	34%/100%	∼34% (combining clinical/ultrasound factors)

The table summarizes, for each study, sample size, assay methodology, proposed basal LH threshold(s), reported sensitivity and specificity, and, when available, the proportion of avoidable GnRH stimulation tests. Assay methodology refers to the analytical platform used in each study.

Abbreviations: CMIA, chemiluminescent microparticle immunoassay; ECLIA, electrochemiluminescence immunoassay; GnRH, gonadotropin-releasing hormone; ICMA, immunochemiluminometric assay; LH, luteinizing hormone; TR-FIA, time-resolved fluoroimmunoassay.

### Strengths and limitations

The main strengths of our study are the relatively large single-center cohort of children evaluated for suspected CPP and the analytical approach used. In particular, we used a censoring-aware statistical method (ROS) to handle the large proportion of bLH values below the assay detection limit. We also assessed both clinically used thresholds and ROC-derived cutoffs, which allowed us to compare statistical performance with potential clinical applicability.

Several limitations should also be acknowledged. The retrospective, single-center nature of the study raises the possibility of referral bias and limits generalizability beyond our clinical setting. Despite applying censoring-aware methods, some uncertainty remains in the interpretation of very low hormone concentrations, particularly near the assay detection limit, where estimates are model-dependent. A further limitation is that the LH assay platform changed during the study period, from Abbott to Roche Cobas, with different limits of detection. However, a sensitivity analysis restricted to the Roche cohort showed findings broadly consistent with those of the overall cohort analysis, suggesting that the main conclusions were not affected by the assay change. Ancillary marker analyses, including estradiol, testosterone, and uterine volume, were also based on smaller subsets.

## Conclusions

Early-morning bLH can be used as a first-line test in the evaluation of suspected CPP, provided that assay-specific thresholds are applied. Values ≥ 1.0 U/L had strong rule-in value in this cohort and may support more selective use of GnRH stimulation testing. Low or indeterminate values still require careful clinical interpretation, particularly in the early stages of pubertal activation, and may still need dynamic testing. Overall, bLH may help make the diagnostic pathway for suspected CPP more efficient while reserving GnRH stimulation testing for selected cases, with potential benefits not only for patient burden but also for the use of healthcare resources and direct costs. Prospective studies are needed to further define assay-specific thresholds and to establish how far it can safely reduce the need for GnRH stimulation testing in routine practice.

## Data Availability

Some or all datasets generated during and/or analyzed during the current study are not publicly available but are available from the corresponding author on reasonable request.
